# Perceived barriers to achieving a healthy weight: a qualitative study using focus groups at public and private schools in Guatemala City

**DOI:** 10.1186/s12889-016-3978-9

**Published:** 2017-01-05

**Authors:** Luisa Madrigal, Inez Adams, Violeta Chacon, Joaquin Barnoya

**Affiliations:** 1 Cardiovascular Surgery Unit of Guatemala Research Department, 5a Av. 6-22 zona 11, Guatemala, 01011 Guatemala; 2 Institute of Nutrition of Central America and Panama, Calzada Roosevelt 6-25 zona 11, Guatemala, 1188 Guatemala; 3 Harvard T. H. Chan School of Public Health, Department of Social and Behavioral Sciences, 401 Park Dr. Room 403-E, Boston, MA 02215 USA; 4 Division of Public Health Sciences, Department of Surgery, Washington University in St. Louis, St. Louis, MO USA

**Keywords:** Overweight, Obesity, Weight perception, Physical activity, Eating

## Abstract

**Background:**

Overweight prevalence among Guatemalan girls is higher in public than in private schools. Little is known about adolescent girls’ perceptions of the right ways to achieve a healthy weight. This study examines public and private school adolescent girls’ perceptions of a “healthy weight,” and barriers and facilitators to achieving it.

**Methods:**

We conducted 4 focus groups in public and private schools in Guatemala City with girls from 13 to 15 years old. The discussion guide included open-ended questions and activities aimed at examining perceptions of “healthy weight” and barriers and motivators to achieving it within the school environment. Focus groups were audio-recorded and transcribed. Data analyses followed established methods of content analysis.

**Results:**

Twenty-eight girls (private school, *n* = 12; public school, *n* = 16) of ages ranging from 13.1 to 15.9 years (median, 14, IQR, 13.6–14.9) participated in the study. Girls identified images of thin and fit women as healthy. They cited healthy eating and physical activity as ways to achieve a healthy weight. Within the school environment, barriers to maintaining a healthy weight included a lack of healthy food options and the prioritization of sports for boys over girls. In public schools, facilities were less than optimal; in private schools, girls’ access to facilities was limited. Public school girls stated that their uniforms were inappropriate for exercising.

**Conclusion:**

Our findings support the need to provide more healthy food options in Guatemalan schools. In addition, physical activity for girls should be promoted and facilities made available for their use.

## Background

Overweight and obesity among adolescents in Latin America have reached epidemic proportions, particularly in urban settings [1, 2]. The combined prevalence of overweight and obesity among adolescents ranges from 16.6% in Colombia to 35.8% in Mexico [1]. In Guatemala, 34.6% of adolescents are overweight or obese [3]. Overweight adolescents are more likely to become overweight adults [4] and therefore more likely to develop non-communicable diseases [5], such as hypertension, diabetes, orthopedic problems, and psychosocial disorders [6, 7].

Overweight prevalence is higher among adolescent girls from lower socioeconomic status (SES) groups [8]. In Guatemala, most adolescents enrolled in public schools are from low SES households, while those in private schools are from higher SES households [9]. In adolescent girls ages 13–15, the combined overweight and obesity prevalence is 42.9% among those in public schools and 35.1% among those in private schools [3]. Understanding the drivers of obesity across gender and SES is key to creating appropriate policy interventions.

Previous research suggests that adolescent girls are capable of accurately judging the quality of their diets [10, 11] and their physical activity levels [12]; however, knowledge of recommended dietary and physical activity guidelines is not sufficient, as girls usually consume whatever food is available, [13, 14] and participation in physical activities is limited to what is locally accessible [15]. Because adolescent girls spend significant amounts of time in school, understanding educational environments may be important to battling the obesity epidemic [16].

Research examining adolescent girls’ knowledge and perceptions of healthy weight has been conducted in high-income countries. For example, among adolescent girls in the United States (US), those who perceived themselves as overweight had worse physical, emotional, and social outcomes than those who perceived themselves as normal weight [17]. Canadian girls associated healthy weight with a combination of healthy eating and regular physical activity [18]. To our knowledge, no studies have gathered data on girls’ perceptions about healthy weight or on school-based facilitators and barriers to achieving a healthy weight in low/middle income countries (LMIC) like Guatemala. This study sought to examine perceptions of “healthy weight” among adolescent girls; the perceived barriers to achieving or maintaining healthy weight; and the differences between private and public schools as a proxy for SES differences.

## Methods

### Study setting

We conducted 4 90-min focus groups with adolescent girls in 2 public and 2 private secondary schools (1 focus group per school) in Guatemala City. School type (i.e., public or private) acted as a surrogate for SES; this classification has previously been used in Guatemalan studies [10]. We used purposeful sampling with the intention of recruiting 8 participants per group, 32 in total. Inclusion criteria were: enrollment in grades 7 through 9 and age 13 to 15 years. At each school, teachers in 4 classrooms per grade were asked to recommend 5 students (adolescent girls) who were articulate and not shy [19]. From the list provided, 8 participants were selected from each grade using a random digit generator. We obtained verbal assent from participants and written informed consent from parents. Approval for this research was obtained from the Institute of Nutrition of Central America and Panama (INCAP) Institutional Review Board before recruitment.

Focus groups were conducted in classrooms so that girls would feel comfortable [19]. The use of focus groups in qualitative research encourages shared discussion between study participants, and is useful when little is known about a topic of interest [20, 21]. The first author along with a research assistant conducted the focus groups. All members of the research team have been trained in conducting focus groups. Participants received a light snack and a library gift card (50 GTQ, approximately $6.50 US) as remuneration for their participation.

### Focus group guide

The research team developed a semi-structured interview guide designed to gather narrative on girls’ perceptions about healthy weight and school-based environmental barriers and motivators to achieving a healthy weight. The guide consisted of 10 open-ended questions and 4 activities created to generate data about how girls defined “healthy” and what they perceived to be a healthy weight (e.g., “What does a healthy person look like?”). The guide also solicited narrative from girls on the best way to achieve and maintain a healthy weight (e.g., “What do people do in order to achieve or maintain a healthy weight?”). Finally, we explored the barriers and motivators to maintaining a healthy weight in the school environment (e.g., “How does your school environment help you achieve or maintain a healthy weight?”).

In order to corroborate the data, 4 activities were conducted. At the time of recruitment, girls were instructed to bring a picture of a “healthy looking woman” to the focus group. The other 3 activities took place during the focus groups. First, after the icebreaker, girls were asked to write down the top 3 things that they thought contributed to good health. For the second and third activities, girls were presented with the pictorial Collins Scale, which includes images of the same woman at 7 different body weights, ranging from thin (image #1) to obese (#7) [22]. Images #3 and #4 are considered as representative of healthy weights. They were asked to select the image on the scale that represented a) the healthiest weight and b) their desired weight.

### Data analysis

Focus groups were audio-recorded and transcribed verbatim. Transcripts were manually coded by 2 team members. Following independent coding, discrepancies were discussed until consensus was achieved. As questions about healthy weight as well as barriers and facilitators to achieving it were approached in an extremely open-ended way, narrative about a specific topic was often discussed during responses to different questions; therefore, a question-by-question analysis was not appropriate for this study. Rather, transcripts were coded for the following major themes: healthy, healthy weight, nutrition, physical activity, school-based facilitators and barriers to healthy weight, and other facilitators and barriers to healthy weight outside of school.

## Results

Twenty-eight girls (12 from private and 16 from public schools) participated in 4 focus groups. Ages ranged from 13.1 to 15.2 years old (median, 13.9, IQR, 13.3–14.5) in private schools and from 13.1 to 15.9 (median, 14.2, IQR, 13.8–15.0) in public schools. Responses to the 3 main topics were similar across school type.

### Perceptions of healthy weight

Participants defined a “healthy weight” as being thin, fit, and strong. They also characterized individuals of “healthy weight” as having proportionate bodies and feeling good about themselves. Participants were asked to bring clippings of a “healthy looking woman” to focus groups. All images portrayed fit, thin, and young women, usually in exercise clothing. Ten clippings included women eating produce, engaged in physical activity, or both. In some of the images, women were holding weights or measuring tape. The remainder of the clippings were still shots or portrayed women doing daily activities (e.g., shopping, working).

After girls were shown the Collins Scale and asked to choose the woman with the healthiest body and the woman whose body matched their desired weight, most girls (75% from private and 81% from public schools) chose image #4 as the healthiest body, while the rest of the girls chose image #3. Most girls also chose image #4 (58.3 and 87.5%, respectively), followed by image #3 as their desired body. Private school girls were more likely to identify their desired body as slightly smaller than the one they identified as the healthiest. None of the participants chose images 1, 2, 5, 6, or 7 as representative of the healthiest or most desired body.

### Ways to achieve a healthy weight

Girls in both school types agreed that a balanced diet and physical activity are the best ways to achieve and maintain a healthy weight. They also thought that balancing food intake and physical activity is key. For example, a public school girl stated, “Avoid eating a lot of junk food, like hamburgers and pizzas; it’s not bad to eat them, but not very often.”

Participants were asked to think of a friend whom they would describe as healthy and to explain why they chose that friend. Girls most often mentioned friends who have balanced diets or eat healthy; exercise often; drink water; and avoid junk food. For example, a private school girl stated, “I would describe [my friend] as healthy because she exercises and has good physical health. She eats healthy; doesn’t eat a lot of junk food; and practices good hygiene and habits.” Another girl said, “She is cheerful. She is in great physical condition and is athletic. She loves to drink water and always eats salads.”

Regarding nutritional knowledge, participants acknowledged that watching portion sizes is important. They mentioned certain nutrients as being integral to a healthy diet and they listed specific foods that contain each one. For example, a private school girl explained, “A balanced diet contains meat (i.e., protein), salad or vegetables, a fruit dessert, juice, and some carbohydrate, like noodles.” A public school girl said, “To be healthy means to eat the 3 basic meals: breakfast, lunch and dinner…. Proper nutrition means having vitamins, proteins, minerals and less fat.” Girls stated that consuming diet pills and skipping meals are unhealthy practices, and some talked about the dangers of bulimia and anorexia. A public school girl explained, “When [some girls] want to be thin, they start taking vitamins to lose weight.... They aren’t even vitamins, but pills. They skip meals; throw up; and do other things that help them lose weight.” Many girls reported that their family members discussed unhealthy weight loss methods with them. One private school girl explained, “My mother tells me, skipping meals is wrong because it makes your metabolism slower and it’s difficult to digest or process the nutrients from food.” In both school types, girls believed that there is a genetic predisposition to being overweight; however, they added that “being disciplined” can help even if you have a genetic predisposition to be overweight.

### Perceived facilitators and barriers to maintaining a healthy weight

Girls were asked to identify the facilitators and barriers to achieving a healthy weight in the school environment. Perceived school-based facilitators included mandatory Physical Education (PE) classes, nutrition education, optional team sports, and sports facilities that are available during recess. In both school types, girls appreciated the fact that PE was a mandatory part of the curriculum. In all focus groups, girls said they attend 2 PE periods per week. Girls in private schools stated that some of their science or home economics teachers “care enough” to teach them about nutrition, although this is a mandatory part of the curriculum.

Private school girls mentioned optional team sports, which require tryouts, as facilitators; however, their options for participating are limited. Another facilitator in private schools is the availability of sport facilities. Girls described them as spacious and safe (i.e., closed private spaces as contrasted with open public spaces), explaining that outside of school it is not uncommon for people to be the victims of crime (i.e., robbery or kidnapping).

Regarding school-based barriers to achieving a healthy weight, girls reiterated that class lectures on nutrition are dependent upon teacher interest. Girls said that teachers who “care” address the topic comprehensively and enthusiastically. For example, a private school girl stated, “Our science teacher pays attention to this topic. About a month ago, we had an activity, in which we had to present a healthy food that we could easily cook.” In contrast, teachers who “do not care” cover the basic topics and do so with indifference.

Another barrier that the girls mentioned was the limited selection of healthy food options available at kiosks on campus. A private school girl explained, “I think the fact that we have kiosks inside [the school] does not help, because it induces us to buy and eat junk food.”

Most girls from both private and public schools said that teachers treat them differently than they do the boys, in regards to physical activity. Teachers are less likely to encourage physical activity for girls. For example, one private school girl commented that teachers play soccer with the boys, but not with the girls. According to the girls, boys often “take over” the best sports facilities and exclude them; this is true for public and private schools. Because this typically happens during recess, teachers are either unaware or chose not to intervene. With regards to optional team sports, boys have more options and receive more support than girls. A private school girl explained, “I went to ask if they could coach a girls’ soccer team, but they said ‘No,’ because the teachers were busy with [boys’] competitions and games.” Another private school girl said, “There is a gym here, but it is more for teachers than students. A boy in my class asked if they [a boys’ sports team] could use the gym for training and they allowed it. But if a girl asks, they will not listen.”

Girls in both private and public schools faced barriers to getting physical activity outside of school. Their primary explanation was that they were simply too busy. A private school girl mentioned, “Being so busy with homework and studying takes time from, for example, exercising or going out. It also makes you stressed and angry. It’s like, ‘Gosh! I have to do this and this!’” Generally, girls in both types of schools felt that they have little control over their schedules. Beyond studying, they expressed that a lot of their time is spent on family obligations (e.g., excessive house chores).

While some participants reported lack of time for physical activity, others expressed a lack of interest. Some of them preferred to spend their leisure time watching TV and interacting on social media. As one public school girl explained, “There are many girls that don’t like playing ball, and if they do, they are only interested for a little while. There are only a few of us that really like playing ball.”

### Differences between public school and private school

In our analysis, we searched for differences between public and private schools. Girls in public schools mentioned that one of the barriers to physical activity is their school uniform, which includes a skirt. Many felt uncomfortable playing while wearing skirts. One public school girl explained the challenge; “Because of the shoes and skirts, we feel uncomfortable. Sometimes the wind lifts our skirts.” Private school girls in our sample had the option of wearing uniform skirts or pants.

Public school girls reported being more affected by their home environment. They identified lack of neighborhood facilities and neighborhood safety as barriers to physical activity and consequently, a healthy weight. One girl stated, “It is not safe to go out around my house because there are many criminals.” Girls added that there are not enough green spaces or “natural environments” where they can enjoy physical activities.

Even though there were some differences between private and public school girls, most facilitators and barriers were reported in both settings. Most barriers were related to environmental and social norms.

## Discussion

In this study we found that girls’ perceptions of healthy and desired body weights did not differ between public and private school girls. Our sample reported more environmental (e.g., availability of sports facilities) and social (e.g., lack of encouragement from teachers) barriers to achieving a healthy weight than personal or knowledge barriers. The main barrier in both school types was the prioritization of physical activity and organized sports for boys over girls.

In Guatemala, most low SES adolescent girls attend public schools while high SES girls are more likely to attend private schools [9]. In Poland, higher SES girls have slightly different perceptions than low SES girls, regarding perceived and desired body weight [23]; in our sample, girls’ opinions were very similar in both settings.

Similar to Canadian girls [18], the girls in our sample were aware of the importance of a healthy diet and physical activity in achieving a healthy weight. They had some knowledge about energy balance, as they mentioned a balance between food intake and physical activity as being key to weight maintenance. Knowledge about nutrition is a facilitator of optimal dietary practices and ideal weight status; however, it must be combined with environmental opportunities (i.e., healthy food and physical activity opportunities) [10, 24, 25].

In our sample, girls did not seem to have distorted perceptions of healthy weight resulting from media influences as has been previously suggested [26]. They identified medium body sizes on the Collins scale as the healthiest and desired weights, consistent with previous studies using rating scales [27].

Adolescents in other studies have identified the absence of healthy food in schools as a barrier to healthy eating [28]. In our study, girls from private and public schools reported that they were regularly tempted to buy the junk food that was available at kiosks because healthy food options were lacking.

According to our findings, mandatory PE classes are facilitators to achieving a healthy weight. This is consistent with findings that Costa Rican adolescents consider comprehensive PE programs to be facilitators to achieving a healthy weight, especially among girls [29]. In many cases, PE is the only opportunity for many low SES adolescents to engage in weekly physical activity [15, 30].

The availability of sport facilities at schools is a facilitator for achieving or maintaining a healthy weight. Sports facilities in private schools are spacious and safe. This was not the case for public schools, which have insufficient facilities [31]. Usually, adolescents from low SES schools have more restricted access to high quality sports facilities compared to adolescents at high SES schools [32]. Girls from private schools mentioned optional team sports as a facilitator. These activities involve supervision and training. However, public school girls do not have access to these activities. Even when facilities are available, gender disparities remain. Girls mentioned that teachers are less likely to encourage physical activity for girls and respond to boys’ requests more readily. They also mentioned that boys “take over” the best facilities for their activities. It is often easier for boys to access and use sports facilities than for girls [30]. In a British study, girls who reported sports-related gender disparities recommended interventions to modify teachers’ attitudes and address stereotypes regarding girls’ sports [33]. Providing opportunities for all students may increase girls’ participation in physical activities. [34].

Previous research suggests that girls’ physical activity practices are affected by personal and societal ideals about gender norms and feminine ideals [35]. Girls in our study reported that one barrier to physical activity was excessive housework. It may be the case that promoting a more equitable distribution of house chores will free up time for girls to participate in physical activities.

Although we did not query girls about neighborhood facilities and safety, these issues were raised among public school girls. Low SES Costa Rican and Mexican adolescents stated that facilities and a supportive social environment would be motivators to physical activity [29, 36]. In addition, neighborhood safety is a barrier commonly mentioned by adolescents in the US, crime activity is poorly associated with physical activity [15].

To our knowledge, this is the first study that explores Guatemalan girls’ perceptions of healthy weight. This was an exploratory qualitative study. The data collected provide information on beliefs about how to achieve a healthy weight and the barriers to doing so. Our findings can be used to inform school interventions and larger qualitative research studies. However, our findings should be interpreted in light of some limitations. First, due to the qualitative nature of this study, our findings can not be generalized outside of the study sample. Second, we did not collect data on participants’ weight as we believed it might influence their answers or willingness to participate. Third, our sample only included adolescents enrolled in school; adolescents not participating in the educational system for different reasons may have different perceptions and face additional and/or unique barriers to achieving a healthy weight. Finally, given the nature of the recruitment process, the sample size was not the same in public and private schools. Nevertheless, this study is valuable as a first approach to understanding girls’ motivators and barriers to achieving a healthy body weight in a LMIC, Guatemala.

## Conclusions

In conclusion, the perceptions of healthy weight in girls are adequate and do not differ by SES in our sample. This suggests that more effort should be given to increasing the availability of healthy food and creating physical activity opportunities than in increasing knowledge. The main barriers to achieving or maintaining a healthy weight are environmental and social and are driven by gender bias. Environmental and social barriers have been found to be as important to achieving a healthy weight as personal or knowledge barriers [37]. Educational interventions or policies should be accompanied by the creation of environmental opportunities. Efforts should be made to create equal opportunities for girls and boys in both private and public Guatemalan schools to participate in physical activity. In addition, access to adequate sports facilities in all schools would facilitate participation in physical activity. Finally, a focus on providing healthy food options in and around schools is necessary.

## Abbreviations

INCAP: Institute of Nutrition of Central America and Panama
LMIC: Low/middle income countries
PE: Physical education
SES: Socioeconomic status
US: United States
